# Molecular Characterization of Community-Associated Methicillin-Resistant *Staphylococcus aureus* in Iranian Burn Patients

**DOI:** 10.30699/ijp.2019.94189.1917

**Published:** 2019-09-22

**Authors:** Samira Tajik, Shahin Najar-Peerayeh, Bita Bakhshi, Reza Golmohammadi

**Affiliations:** 1 *Department of Bacteriology, Faculty of Medical Sciences, Tarbiat Modares University, Tehran, Iran*; 2 *Molecular Biology Research Center, Systems Biology and Poisonings Institute, Baqiyatallah University of Medical Sciences, Tehran, Iran*

**Keywords:** Burn, Patients, MRSA, SCC*mec*

## Abstract

**Background & Objective:**

Methicillin-resistant *Staphylococcus aureus* (MRSA) is reported as one of the important bacterial causes of burn wound infections. This study was carried out to investigate molecular characterization of community-associated MRSA (CA-MRSA) isolated from Iranian burn patients.

**Methods:**

A total of 31 isolates of *S. aureus* were collected from the Motahari Burns Hospital (Tehran, Iran) in 2016. All isolates were collected from outpatients and inpatients within 48 hours of admission. The *mecA*, *pvl*, *tsst-1, hla-α, *and* psmα *genes detecting, SCC*mec*, *agr *and PFGE typing were done.

**Results:**

A total of 13 (41.9%) isolates were cefoxitin-resistant and *mecA*-positive, which were considered as MRSA. The SCC*mec* typing MRSA strains revealed type II in 1 (7.7%), type III in 9 (69.2%), and other types in 3 isolates (23.7%) cases. The* agr* typing of all 31 isolates showed that 14 (45.2%), 1 (3.2%), 6 (19.4%), and 10 (32.3%) strains belonged to *agr* groups 1, 3, 4, and unknown type, respectively. The *pvl*, *tsst-1, hla-α, *and* psmα *genes were positive in 3 (9.7%), 4 (12.9%), 21 (67.7%), and 31 (100%) isolates, respectively. Considering the cut-off values of ≥50%, 3 groups of related isolates (cluster A1, B1, and C1) in PFGE study were observed.

**Conclusion:**

The MRSA strains of this study were initially isolated as Community-associated S. aureus (CA-MRSA); however molecular characterization showed that a significant proportion of them had hospital-associated MRSA (HA-MRSA) features. Therefore, it is likely that the HA-MRSA strains are spread among the community.

## Introduction

Due to the skin damaged and weakened innate immunity, burn patients become susceptible to various infections (1, 2), among which bacterial infections are the main problems in hospitalized burn patients (3, 4). 

*Staphylococcus aureus* is one of the most common bacteria isolated from burn infections (5). Methicillin-resistant *S. aureus* (MRSA) including hospital-associated MRSA (HA-MRSA) (3, 6) and community-associated MRSA (CA-MRSA) (7) are reported as the important bacterial causes of burn wound infections. The centers for disease control (CDC) defined a CA-MRSA infection as any MRSA infection diagnosed for an outpatient or within 48 hours of hospitalization if the patient lacks such healthcare-associated MRSA risk factors as dialysis or use of invasive medical devices, surgery, and residence in a long-term care facility or hospitalization during the previous year; all other MRSA infections were considered to be HA-MRSA (8-10).

Cefoxitin is a potential inducer of the *mecA *regulatory system and is used for screening MRSA strains (11). Staphylococcal cassette chromosome *mec* (SCC*mec*) element of MRSA strains carry *mecA* gene that mediates resistance to methicillin (10, 12). HA-MRSA strains carry large SCC*mec* types II and III, which are responsible for resistance to other antibiotics. However, SCC*mec* types IV and V elements are found in CA-MRSA strains (10, 13).

It should be noted that there are several problems in terms of using CA-MRSA and HA-MRSA. First, both strains can be circulated in the community; second, it is possible that CA-MRSA strains are gradually converted to nosocomial pathogens (10, 14). 

The pathogenicity of *S. aureus* is associated with various virulence factors genes including accessory gene regulator (*agr*) system, panton-valentine leukocidin (*pvl*), toxic shock syndrome toxin (*tsst*), α-hemolysin (*hla*), and alpha-type phenol soluble modulins (*psmα*). These virulence factors can make extensive infection and severe damage (7, 12, 15, 16). The *agr* regulatory system has a crucial role in the development of skin infection cases by most CA-MRSA USA300 strains (17). The PVL toxin can be found in both MSSA and MRSA strains (18) but typically is associated with CA-MRSA strains (7). TSST-1 is a superantigen that is absorbed from cutaneous infections and it localizes the kidneys (19). It is reported that the pore-forming α-hemolysin of *S. aureus* plays a key role in soft tissue infection (20). The *psmα* may play a role in biofilms development and lysis of human cell types such as leukocytes and erythrocytes; also, it stimulates inflammation response (21).

Phenotypic and genotypic characterization of CA-MRSA and HA-MRSA can be helpful in the treatment, infection control, and prevention of the spread of antibiotic-resistant bacterial strains (1, 22, 23). This study was carried out to investigate molecular characterization of CA-MRSA isolated from Iranian burn outpatients and hospitalized patients within 48 hours of admission. 

## Materials and Methods

**Bacterial Isolates**

A total of 31 isolates of *S. aureus* were collected from Shahid Motahari Burns Hospital (Tehran, Iran) in the time period of May to October 2016. All isolates were collected from outpatients and inpatients within 48 hours of admission. Bacterial morphology study and biochemical tests including catalase, tube and slide coagulase, DNase, and mannitol-fermenting were done to identify *S. aureus*. After identification, all strains were preserved in trypticase soy broth medium containing 15% glycerol at -70°C.

**Antimicrobial Susceptibility Testing**

Antimicrobial susceptibility testing was done by disk diffusion method according to the clinical and laboratory standards institute (CLSI) 2015 guidelines. Antibiotic discs containing cefoxitin (30 μg), gentamicin (10 μg), erythromycin (15 μg), tetracycline (30 μg), rifampin (5 μg), clindamycin (2 μg), ciprofloxacin (5 μg), trimethoprim-sulfamethoxazole (1.25/23.75 μg), and linezolid (30 μg) (MAST UK) were used. *S. aureus* ATCC 25923 was used as a control strain.

**Molecular Detection and Typing**

**DNA Extraction**

Four to five fresh colonies of bacteria were suspended in 400 μL of lysis solution buffer containing lysostaphin (200μg/mL) (Sigma-Aldrich Co, Germany). Then, DNA was extracted by phenol/chloroform /isoamyl alcohol (Merck Co).

**PCR Amplification**

The *mecA* was used as target gene for the detection of MRSA strains (24). The primers described by Zhang *et al.* (24) and Shopsin *et al.* (25) were used for SCC*mec* typing and *agr* typing, respectively. RN6390, RN6607, RN8465, and RN4550 strains were used as positive control for *agr* typing. The *pvl*, *tsst-1, hla-α *(26), and* psmα *(27) genes were used as target gene for virulotyping. The list of primers sequences of this study, target genes, and the length of PCR products are presented in Table 1.

**Table1:** The list of primers sequences for *mecA, pvl*, *tsst-1, hla-α, *and* psmα *genes detection, SCC*mec *and* agr* typing and PCR products length

Ref	Size (bp)	Sequence (5ʹ→3ʹ)	Primer	
(24)	146	F: GTGAAGATATACCAAGTGATT R: ATGCGCTATAGATTGAAAGGA	*mecA*	*mecA* detection
(24)	613	F: GCTTTAAAGAGTGTCGTTACAGG R: GTTCTCTCATAGTATGACGTCC	SCC*mec *I	SCC*mec* typing
	398	F: CGTTGAAGATGATGAAGCG R: CGAAATCAATGGTTAATGGACC	SCC*mec *II	
	280	F: CCATATTGTGTACGATGCG R: CCTTAGTTGTCGTAACAGATCG	SCC*mec *III	
	776	F: GCCTTATTCGAAGAAACCG R: CTACTCTTCTGAAAAGCGTCG	SCC*mec *IVa	
	493	F: TCTGGAATTACTTCAGCTGC R: AAACAATATTGCTCTCCCTC	SCC*mec *IVb	
	200	F: ACAATATTTGTATTATCGGAGAGC R: TTGGTATGAGGTATTGCTGG	SCC*mec *IVc	
	881	F: CTCAAAATACGGACCCCAATACA R: TGCTCCAGTAATTGCTAAAG	SCC*mec *IVd	
	325	F: GAACATTGTTACTTAAATGAGCG R: TGAAAGTTGTACCCTTGACACC	SCC*mec *V	
(25)	-	ATGCACATGGTGCACATGC	panF	*agr *typing
	440	GTCACAAGTACTATAAGCTGCGAT	*agr *I-R	
	572	GTATTACTAATTGAAAAGTGCCATAGC	*agr *II-R	
	406	CTGTTGAAAAAGTCAACTAAAAGCTC	*agr *III-R	
	588	CGATAATGCCGTAATAC CCG	*agr *IV-R	
(26)	502	F: GGAAACATTTATTCTGGCTATAC R: CTGGATTGAAGTTACCTCTGG	*pvl*	Virulence genes typing
	398	F: TTATCGTAAGCCCTTTGTTG R: TAAAGGTAGTTCTATTGGAGTAGG	*tsst-1*	
	744	F: CGGTACTACAGATATTGGAAGC R: TGGTAATCATCACGAACTCG	*hla-α*	
(27)	176	F: TATCAAAAGCTTAATCGAACAATTC R: CCCCTTCAAATAAGATGTTCATATC	*psma*	

The final volume of 25 mL PCR reaction mixture containing reverse and forward primer (10 pmol) (Pishgam Biotech Co, Iran), DNA taq polymerase enzyme (1.5 units), dNTPs (0.02 mM), MgCl_2_ (0.1 mM), buffer (Fermentase Co, Lithuania), deionized distilled water, and DNA template were used for PCR amplification. The PCR amplification performed in thermo cycler (BIO RAD T100) by following annealing temperatures: *mecA; *55°C, SCC*mec* typing; 60°C, *agr *typing; 50°C, and virulence genes typing; 50°C for *pvl and hla-α*, and 51°C for *tsst-1 *genes. 

**Pulsed-field Gel Electrophoresis (PFGE)**

PFGE analysis based on *Sma*I restriction enzyme (Takara) digestion was carried out using the PulseNet standard protocol on 13 *SCCmec*-positive *S. aureus* strains (PulseNet, www.cdc.gov/pulsenet). After optimizing conditions, PFGE was run on CHEF MAPPER^XA^ (BIO RAD) device as follows: initial switch 5 s, final switch 40 s, run time 21 h, voltage 200 (6v/cm). 1% agarose gel (intron biotechnology) in TBE 0.5 X was used for electrophoresis. According to the PulseNet protocol, *Salmonella enterica *serotype Braenderup (H9812) was used as a control. After gel staining with ethidium bromide (10 mg/mL), imaging was performed by UV light gel documentation system (BioDocAnalyze). GelCompar II version 6.6.11 software was used for drawing dendrogram graph and interpretation of results. Cut-off values of ≥50% were considered to define clusters.

## Results

**Patients**

A total of 31 non-duplicate *S. aureus* isolates were investigated in this study. All isolates were collected from burn patients within 48 hours of admission. 20 (64.5%) of these patients were inpatients and 11 (35.5%) outpatients.

**Antimicrobial Susceptibility Testing**

Antibiotic susceptibility test results revealed that a total of 13 (41.9%) isolates were resistant to cefoxitin, which were considered as MRSA. All isolates were susceptible to Linezolid. The resistance rates of other antibiotics were as follows: erythromycin 15 (48.4%), tetracycline 11 (35.5%), ciprofloxacin 11 (35.5%), gentamicin 10 (32.3%), clindamycin 9 (29.0%), rifampin 6 (19.4%), and trimethoprim-sulfamethoxazole 4 (12.9%) (Table 2). 

**Table2:** Antibiotic resistance of MSSA and MRSA isolated from burn patient

Antibiotics	MSSA (n=18)	MRSA (n=13)	Total (n=31)
cefoxitin	0	13 (100%)	13 (41.9%)
erythromycin	6 (33.3%)	9 (69.2%)	15(48.4%)
tetracycline	3 (16.7%)	8 (61.5%)	11(35.5%)
ciprofloxacin	2 (11.1%)	9 (69.2%)	11(35.5%)
gentamicin	1 (5.5%)	9 (69.2%)	10(32.3%)
clindamycin	2 (11.1%)	7 (53.8%)	9(29.0%)
rifampin	1 (5.5%)	5 (38.5%)	6(19.4%)
trimethoprim-sulfamethoxazole	0	4 (30.8%)	4(12.9%)
Linezolid	0	0	0

**Molecular Detection and Typing**

Molecular studies showed that 13 (41.9%) isolates were *mecA*-positive (Table 3). The SCC*mec* typing MRSA strains revealed type II in 1 (7.7%), type III in 9 (69.2%), and other types in 3 isolates (23.1%) (Table 3).

The* agr* typing of all 31 isolates showed that 14 (45.1%), 1 (3.2%), 6 (19.4%), and 10 (32.3%) strains belonged to *agr* groups 1, 3, 4, and unknown type, respectively (Table 3).

The *pvl*, *tsst-1, hla-α, *and* psmα *genes were positive in 3 (9.7%), 4 (12.9%), 21 (67.7%), and 31 (100%), respectively.

**Figure1:**
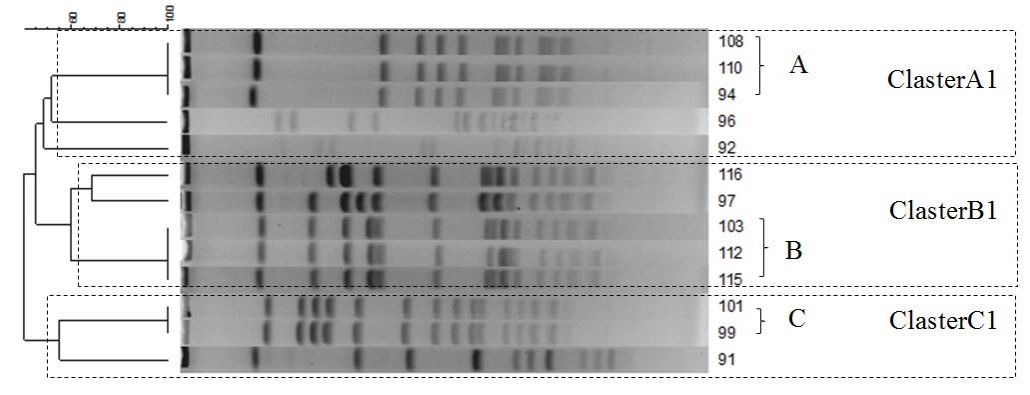
PFGE analysis of CA-MRSA in current study

All 13 MRSA isolates were studied by PFGE. Considering the cut-off values of ≥50%, 3 groups of isolates (cluster A1, B1, and C1) were observed in this study: cluster A1; 3 pulse types, cluster B1; 3 pulse types and cluster C1; 2 pulse types (Figure 1).

**Table 3:** The *mecA, pvl*, *tsst-1, hla-α, *and* psmα *genes detection, and SCC*mec *and* agr* typing result (n=31)

***psma***	*hla-a*	*tsst-1*	*pvl*	*agr*		SCC*mec*		*mecA*	gene
**31(100%)**	21(67.7%)	4(12.9%)	3(9.7%)	14(45.1%)	type 1	9(69.2%)	type3	13(41.9%)	**Positive**
				1(3.2%)	type 3	1(7.7%)	type2		
				6(19.4%)	type 4	3(23.1%)	Not typeable		
				10(32.3%)	Not typeable				
**0(0%)**	10(32.3%)	27(87.1%)	28(90.3)	0(0%)		18		18(58.1%)	**Negative**

## Discussion

*S. aureus* is one of the common causes of burn wound infections (5). Drug-resistant strains of this organism such as MRSA can complicate the treatment process (3, 6, 7). In addition, the strains containing virulence genes such as *agr*, *pvl*, *tsst-1*, *hla-α,* and *psmα* can make extensive infection and severe damage (7, 15, 16, 28, 29).

In current study 41.9% isolates were *mecA*-gene positive and cefoxitin-resistant, so were considered as MRSA. Based on the molecular detection of *mec* gene in different studies, MRSA strains have been reported as 86.4% (30), 88.6% (31), 81.2% (32), and 87.4% (18) in Iran and 55.3% in China (3).

According to the antibiotic susceptibility testing of all 31 strains, the highest resistance was related to erythromycin (48.4%) compared to 58.2% (2), 77.8% (30), and 90.6% (16) in Iran. All the 13 MRSA strains in this study were sensitive to linezolid, which was similar to the findings of the studies conducted by Montazeri (31, 32), and Goudarzi (16) from Iran and Murray (7) from the US. 

The SCC*mec* typing of 13 MRSA strains showed that 9 (69.2%) strains belonged to SCC*mec* III, that's almost is consistent with some studies in Iran; 56.8% (2), 47.5% (33), 70.1% (15), and 71.7% (16) did not match the other studies in Iran; 95.7% (30), China; 95.4% (16), Brazil; 94.8% (34). 7.7% of the these 13 strains belonged to SCC*mec* II, compared with 10% (33) and 2.5% (15) studies in Iran, and (1%) in Brazil (34). Although 3 (23.1%) other types were not detected in this study, SCC*mec* I, IV, and V have been detected in other studies (2, 3, 15, 16, 34). 

Based on the CDC definition of CA-MRSA and HA-MRSA strains (8-10) and according to other studies (10, 13), it seems that most of these SCC*mec* types belonged to HA-MRSA strains. Since sampling was done within 48 hours after admission, the strains were expected to be CA-MRSA. According to a hypothesis, this has been affected by various factors such as having the same source of infection or spread of infected patients with HA-MRSA strains among the community. Since checking the patients’ referral records during the last year was not possible in this study, it is possible that most of these strains were HA-MRSA. Furthermore, having high relative resistance to selected antibiotics including clindamycin in most strains also confirms this probability. Eight (61.5%) of MRSA strains in this study and 75.5% in Goudarzi’s study (16) were resistant to clindamycin, which indicates these strains had high resistance to other antibiotics. Also, 5 (38.5%) clindamycin-susceptible strains in this study had a lower resistance to other antibiotics. Therefore, it can be concluded that 8 (61.5%) of these strains correspond to HA-MRSA and 5 (38.5%) to CA-MRSA.

The PFGE cluster results with cut-off values of ≥50% indicated that MRSA strains in this study, to some extent, had CA-MRSA characteristics. Also, the 3 strains in clusters A1, 3 in B1, and 2 in C1 have the same pattern. This similarity suggests that the source of infection has been the same in each cluster. 

The PVL toxin typically is associated with CA-MRSA strains (7). In the present study, 3 (9.7%) MRSA strains were *pvl*-positive. In contrast to the study by Goudarzi that reported the positive *pvl* strains belonged to SCC*mec* type IV (16), in this study 2 of 3 strains belonged to the SCC*mec* type III. Although all MSSA strains in this study were *pvl*-negative, some other studies in Iran reported that 7.23% (18), 15.1% (16), and 33.3% (18) of MSSA cases were positive.

The *agr* typing assay of all 31 isolates showed that the highest rate belonged to *agr* type 1 (45.1%) compared to 84.9% in Iran (16) and 96.5% in China (3); meanwhile, the lowest rate belonged to *agr* type 3 (3.2%) compared to 15.1% in Iran (16). In this study, 32.3% strains were not typeable, which might be related to the presence of different mutations, sensitivity, and specificity of primers.

The *tsst-1* gene detection showed that 12.9% of the isolates in this study belonged to MSSA strains, but this gene was not seen in MRSA strains. In contrast to this study, in a different study in Iran 18.9% of MRSA isolates were *tsst*-positive which were distributed among SCC*mec* type III (16).

In the present study, 67.7% strains were *hla*-positive compared to another study in Iran which reported 51.8% (15). Data analysis showed that 32.2% belonged to MRSA and 35.5% to MSSA. 

Our results showed that 41.9% of isolates were MRSA, which indicates the presence of antibiotic resistance elements in a significant number of strains and necessity of performing antibiogram before starting treatment. Furthermore, the MRSA strains of this study were initially isolated as CA-MRSA, however molecular characterization showed that a significant proportion of them had HA-MRSA features. Therefore, it is likely that the HA-MRSA strains are spread among the community. Finally, the investigation of virulence factors in this study showed the presence of various toxin genes in isolates that can enhance their ecological niche ability and also make treatment more complicated, especially in burn patients.
